# miR-214 Promotes Aggressive Behavior in Triple-Negative Breast Cancer by Functionally Targeting the 3′-UTR of FRK

**DOI:** 10.3390/biomedicines14050971

**Published:** 2026-04-23

**Authors:** Serin Moon, Sooeun Oh, Dong-Min Kim, Jieun Lee, Ahwon Lee

**Affiliations:** 1 Department of Hospital Pathology, Seoul St. Mary’s Hospital, College of Medicine, The Catholic University of Korea, Seoul 06591, Republic of Korea; tptpfls1239@naver.com (S.M.); dhtndms2@naver.com (S.O.); snoopy08246@naver.com (D.-M.K.); 2 Division of Medical Oncology, Department of Internal Medicine, Seoul St. Mary’s Hospital, College of Medicine, The Catholic University of Korea, Seoul 06591, Republic of Korea; 3 Cancer Research Institute, The Catholic University of Korea, Seoul 06591, Republic of Korea

**Keywords:** breast cancer, triple-negative breast cancer, microRNA-214, FRK, tumor suppressor, invasion

## Abstract

**Background/Objectives**: MicroRNAs (miRNAs) are key regulators of gene expression and have been implicated in multiple aspects of cancer progression. However, the role of miR-214-3p in breast cancer remains controversial. In this study, we investigated the functional role of miR-214-3p and explored its potential regulatory target in breast cancer, particularly in triple-negative breast cancer (TNBC). **Methods**: miR-214-3p expression was evaluated in breast cancer cell lines. Luciferase reporter assays were performed to assess functional targeting of the FRK 3′-UTR. Functional assays, including proliferation, migration, and invasion assays, were conducted following miR-214-3p overexpression or FRK silencing. **Results**: miR-214-3p was markedly upregulated in TNBC cells (MDA-MB-231), while Fyn-related kinase (*FRK*), a potential tumor suppressor, showed an inverse expression trend. Luciferase reporter assays demonstrated that miR-214-3p functionally targets the 3′-UTR of FRK. Functional analyses revealed that overexpression of miR-214-3p significantly increased cell proliferation, migration, and invasion. Notably, silencing of FRK recapitulated these effects, supporting its role as a functional mediator of miR-214-3p. **Conclusions**: This study identifies a miR-214–FRK regulatory axis in breast cancer and suggests its contribution to aggressive tumor behavior. Targeting miR-214-3p or modulating FRK activity may represent a potential therapeutic strategy.

## 1. Introduction

Breast cancer is the most frequently diagnosed cancer in women worldwide and continues to be a major cause of cancer-related death [1]. Among its subtypes, triple-negative breast cancer (TNBC), which lacks expression of estrogen receptor, progesterone receptor, and HER2, accounts for approximately 10–20% of cases and is associated with aggressive clinical behavior, a high rate of metastasis, and limited therapeutic options [2,3]. Therefore, identifying novel molecular mechanisms underlying TNBC progression is critical for the development of effective targeted therapies.

MicroRNAs (miRNAs) are short non-coding RNA molecules that regulate gene expression at the post-transcriptional level by binding to the 3′-untranslated region (3′-UTR) of target mRNAs [4,5]. Abnormal expression of miRNAs has been linked to multiple processes involved in cancer development, including cell proliferation, migration, invasion, and metastasis [4,6].

In particular, recent studies have highlighted the role of miRNAs as central regulators of key oncogenic signaling pathways. These regulatory effects involve major signaling pathways, such as PI3K/AKT, Wnt/β-catenin, and p53 pathways, thereby influencing cell proliferation, survival, metastasis, and therapeutic resistance [7,8].

Among these, miR-214-3p has been reported to play diverse roles in multiple cancers, including breast cancer. Previous studies have demonstrated that miR-214-3p regulates key signaling pathways by targeting genes such as PTEN, β-catenin, and p53 [9,10,11,12]. However, its role in breast cancer remains controversial, with conflicting evidence suggesting both oncogenic and tumor-suppressive functions [9,11,13]. These discrepancies may reflect tumor heterogeneity and subtype-specific effects, particularly in aggressive subtypes such as TNBC.

FRK is a non-receptor tyrosine kinase that has been suggested to exert tumor-suppressive functions in multiple cancer types, including breast cancer [14,15]. Mechanistically, FRK has been shown to interact with and stabilize PTEN by inhibiting its ubiquitination, thereby suppressing PI3K/AKT signaling and regulating cell proliferation and survival pathways [14,16]. Despite these tumor-suppressive functions, the upstream regulatory mechanisms controlling FRK expression in breast cancer remain incompletely understood. Given the tumor-suppressive role of FRK and the reported dysregulation of miR-214-3p in breast cancer, a potential regulatory interaction between miR-214-3p and FRK warrants investigation.

Given the reported dysregulation of miR-214-3p in cancer, we hypothesized that miR-214-3p may contribute to breast cancer progression by functionally targeting the 3′-UTR of FRK. In this study, we investigated the expression and functional role of miR-214-3p in breast cancer cell lines and evaluated its functional targeting of FRK. Our findings provide insight into a miR-214–FRK regulatory axis that contributes to aggressive tumor behavior, particularly in TNBC.

## 2. Materials and Methods

### 2.1. Cell Lines and Cell Culture

The human breast epithelial cell line MCF-10A (ATCC^®^ CRL-10317™) and breast cancer cell lines MCF-7 (ATCC^®^ HTB-22™), MDA-MB-231 (ATCC^®^ HTB-26™), and MDA-MB-468 (ATCC^®^ HTB-132™) were obtained from the American Type Culture Collection (ATCC, Manassas, VA, USA). HEK293, MCF-7, and MDA-MB-231 cells were used for luciferase reporter assays.

MCF-10A cells were cultured in MEGM medium (Lonza, Basel, Switzerland) supplemented with the BulletKit™. MCF-7 cells were maintained in RPMI-1640 medium (Corning, Corning, NY, USA) supplemented with 10% fetal bovine serum (FBS) and antibiotics (100 U/mL penicillin and 100 μg/mL streptomycin). MDA-MB-231 and MDA-MB-468 cells were cultured in Leibovitz’s L-15 medium supplemented with 10% FBS and antibiotics. HEK293 cells were cultured in Dulbecco’s modified Eagle’s medium (DMEM) with 10% FBS and antibiotics.

Cells were maintained at 37 °C under conditions recommended by the supplier.

### 2.2. Selection of Target Gene

Potential target genes of miR-214-3p were predicted using publicly available miRNA databases, including miRTarBase, miRsearch V3.0, and miRNAMap. Among the predicted targets, Fyn-related kinase (FRK) was selected for further analysis based on its reported tumor suppressor function.

### 2.3. miRNA Mimics and siRNA Transfection

Synthetic miR-214-3p mimics, mutant mimics (miR-214-3pm), small interfering RNA targeting FRK (si-FRK), and scrambled control oligonucleotides were purchased from Genolution Pharmaceuticals (Seoul, Republic of Korea). The sequences were as follows: miR-214-3p mimic: 5′-ACAGCAGGCACAGACAGGCAGU-3′; miR-214-3pm (mutant): 5′-AGUCGAGGCACAGACAGGCAGU-3′; si-FRK: 5′-GAUCAGAUGCAGAGAAACAUU-3′; Scrambled control: 5′-UUUUAACUCAGUAUUUUUA-3.

Cells were plated 24 h before transfection and subsequently transfected using Lipofectamine™ 2000 (Invitrogen, Carlsbad, CA, USA) following the manufacturer’s protocol. Oligonucleotides were used at a final concentration of 50 nM. After 4–6 h, the medium was replaced with fresh complete medium, and cells were harvested 48 h post-transfection. Transfection efficiency was confirmed by qRT-PCR, demonstrating increased miR-214-3p expression following miR-214 mimic transfection and decreased FRK mRNA expression following si-FRK transfection. MDA-MB-468 cells were included in the initial expression analysis but were not used in subsequent functional or luciferase assays due to low transfection efficiency and variable experimental responses.

### 2.4. Luciferase Reporter Assay

The 3′-UTR of FRK containing predicted miR-214-3p binding sites was cloned into the psiCHECK-2 vector (Promega, Madison, WI, USA). Mutations in the seed regions were introduced using site-directed mutagenesis.

HEK293, MCF-7, and MDA-MB-231 cells were seeded in 96-well plates (5 × 10^3^ cells/well) and co-transfected with luciferase reporter constructs (20 ng) and miR-214-3p mimics (10 nM). After 48 h, luciferase activity was measured using the Dual-Glo™ Luciferase Assay System (Promega, Madison, WI, USA). Renilla luciferase activity was normalized to firefly luciferase activity. All experiments were performed under identical conditions across the three cell lines.

### 2.5. Quantitative Real-Time PCR (qRT-PCR)

Total RNA was isolated using TRIzol reagent (Thermo Fisher Scientific, Waltham, MA, USA) following the manufacturer’s protocol. Complementary DNA (cDNA) was synthesized using M-MLV reverse transcriptase.

qRT-PCR was carried out using SYBR Green-based detection on a LightCycler 96 system (Roche, Basel, Switzerland). *GAPDH* and *U6* were used as internal controls. Relative expression levels were calculated using the comparative Ct (2^−ΔΔCt^) method [17].

### 2.6. Cell Proliferation Assay

Cell proliferation was evaluated using an MTS-based assay (CellTiter 96^®^ AQueous One Solution, Promega). Cells were plated in 96-well plates (200 μL medium per well) and transfected as described above. At 24, 48, and 72 h post-transfection, MTS reagent was added to each well and incubated for 4 h at 37 °C. Absorbance at 490 nm was measured using a microplate reader.

### 2.7. Migration and Invasion Assays

Cell migration and invasion were evaluated using Transwell chambers with 8 μm pore size polycarbonate membranes (Corning, Corning, NY, USA).

For migration assays, cells were seeded in serum-free medium in the upper chamber, and medium containing 20% FBS was added to the lower chamber. For invasion assays, the upper chamber was pre-coated with Matrigel according to the manufacturer’s instructions.

After 24 h incubation, cells that migrated or invaded through the membrane were fixed with 3.7% formaldehyde, stained with crystal violet for 20 min, and counted under a microscope. Representative images were acquired under identical magnification and imaging conditions.

### 2.8. Statistical Analysis

All experiments were performed at least three times independently (biological replicates). Data are presented as mean ± standard deviation (SD). Statistical analysis was conducted using GraphPad Prism version 9 (GraphPad Software, San Diego, CA, USA). For comparisons between two groups, an unpaired two-tailed Student’s *t*-test was used. For comparisons among three or more groups, one-way analysis of variance (ANOVA) followed by Tukey’s multiple comparisons test was applied. The sample size (n) for each experiment is indicated in the corresponding figure legends. Data were assumed to be approximately normally distributed. A *p*-value < 0.05 was considered statistically significant.

## 3. Results

### 3.1. miR-214-3p Is Upregulated and Shows an Inverse Trend with FRK in TNBC Cells

**Figure 1:**
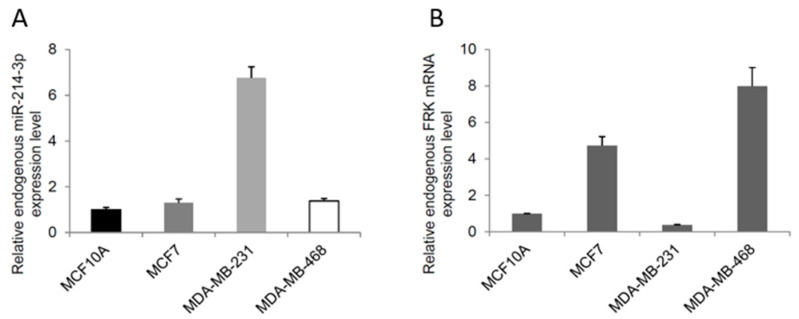
Expression of miR-214-3p and *FRK* in breast cancer cell lines. (**A**) Relative expression levels of miR-214-3p in MCF-10A, MCF-7, MDA-MB-231, and MDA-MB-468 cells, as determined by quantitative real-time PCR (qRT-PCR). (**B**) Relative mRNA expression levels of *FRK* in the same cell lines, measured by qRT-PCR. Expression levels were normalized to *U6* (for miR-214-3p) and *GAPDH* (for *FRK*) and calculated using the 2^−ΔΔCt^ method. Data are presented as mean ± standard deviation (SD) from three independent experiments (*n* = 3).

We initially assessed the endogenous expression levels of miR-214-3p in breast cancer cell lines representing different molecular subtypes. miR-214-3p expression was markedly increased in MDA-MB-231 cells compared to MCF-10A and MCF-7 cells (Figure 1A).

*FRK* mRNA expression showed a partially inverse pattern, with relatively low expression in MDA-MB-231 cells and higher expression in MCF-7 and MDA-MB-468 cells (Figure 1B). Overall, miR-214-3p expression showed an inverse pattern with *FRK* expression, particularly in TNBC cells. However, this relationship was not strictly consistent across all cell lines, suggesting that FRK expression may be regulated by additional factors beyond miR-214-3p.

### 3.2. miR-214-3p Functionally Targets the 3′-UTR of FRK

**Figure 2:**
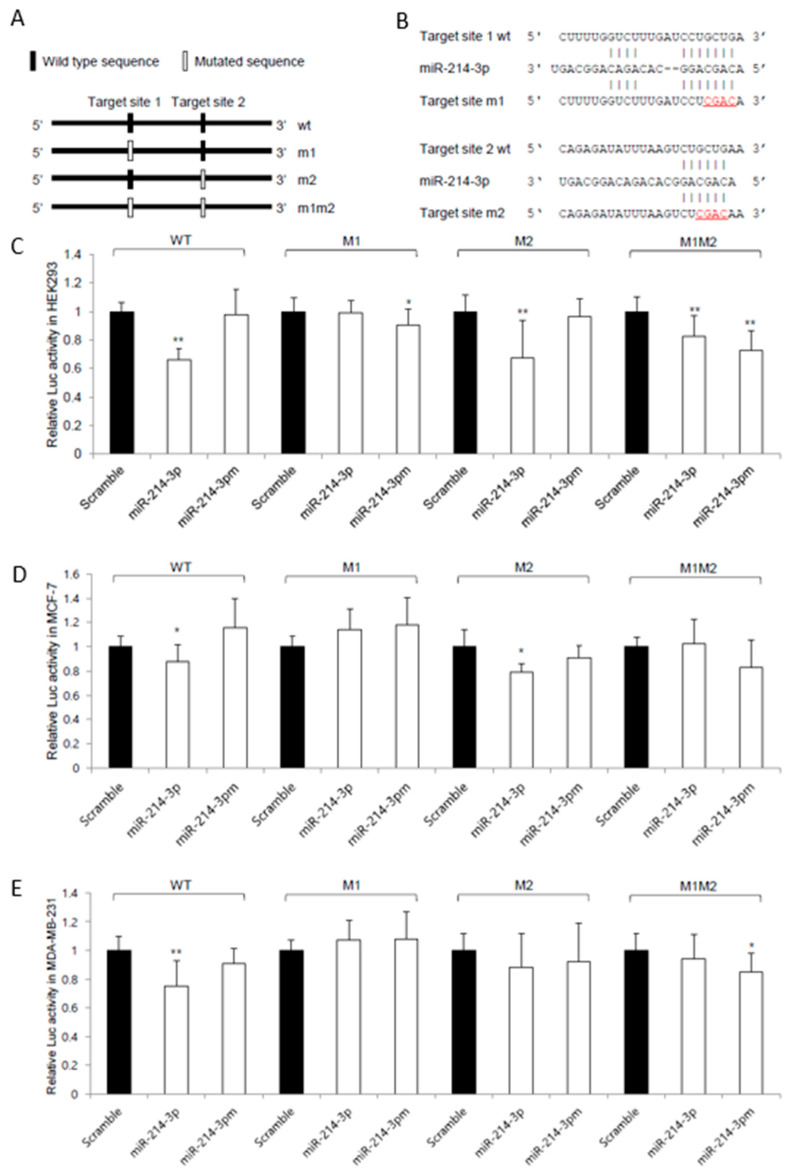
miR-214-3p functionally targets the 3′-UTR of FRK. (**A**) Schematic representation of the predicted miR-214-3p binding sites within the FRK 3′-UTR. Wild-type (WT) and mutant (M1, M2, and M1M2) constructs were generated by altering the seed regions of the predicted target sites. (**B**) Sequence alignment of miR-214-3p with the wild-type and mutant target sites in the FRK 3′-UTR, showing complementary binding and disrupted pairing in mutant constructs. (**C**–**E**) Luciferase reporter assays of wild-type and mutant FRK 3′-UTR constructs in HEK293 (**C**), MCF-7 (**D**), and MDA-MB-231 (**E**) cells co-transfected with miR-214-3p mimics or control oligonucleotides. Mutation of target site 1 markedly attenuated the suppressive effect of miR-214-3p, whereas mutation of target site 2 showed a lesser effect. Relative luciferase activity was normalized to Renilla/firefly luciferase activity. Data are presented as mean ± standard deviation (SD) from three independent experiments (*n* = 3). * *p* < 0.05, ** *p* < 0.01.

To identify potential binding sites of miR-214-3p in the FRK 3′-UTR, bioinformatic prediction analysis was performed. Two putative binding sites were identified, and schematic representations of wild-type and mutant constructs are shown (Figure 2A). Sequence alignment showed complementary binding between miR-214-3p and the predicted target sites (Figure 2B).

To evaluate whether miR-214-3p directly interacts with the FRK 3′-UTR, luciferase reporter assays were conducted using constructs containing the FRK 3′-UTR. Overexpression of miR-214-3p significantly reduced luciferase activity in HEK293, MCF-7, and MDA-MB-231 cells compared to control groups (Figure 2C–E).

To further validate sequence-specific interaction, mutant constructs were generated by altering the predicted seed regions. Mutation of target site 1 abolished the suppressive effect of miR-214-3p, whereas mutation of target site 2 had a lesser effect (Figure 2C–E), indicating that target site 1 is the primary functional site mediating the interaction. These findings indicate that miR-214-3p functionally targets the 3′-UTR of FRK and contributes to the suppression of its expression.

### 3.3. miR-214-3p Promotes Cell Proliferation, Migration, and Invasion via Suppression of FRK

**Figure 3:**
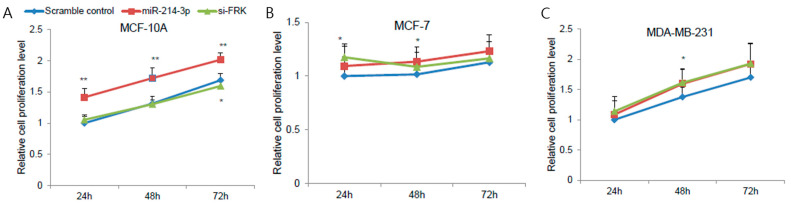

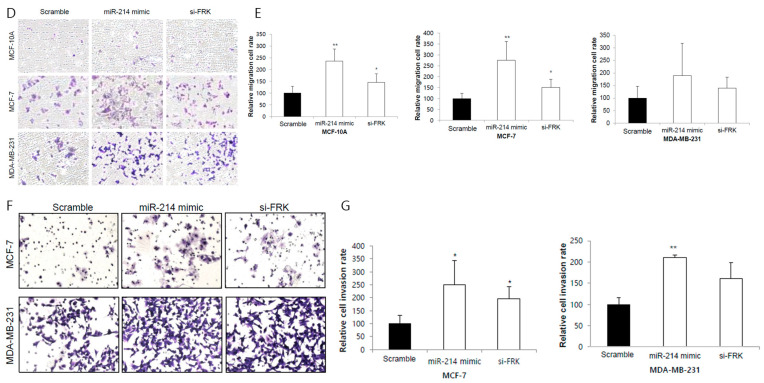
miR-214-3p promotes cell proliferation, migration, and invasion through suppression of FRK. (**A**–**C**) Time-course cell proliferation assays measured at 24, 48, and 72 h using an MTS assay in MCF-10A (**A**), MCF-7 (**B**), and MDA-MB-231 (**C**) cells transfected with scramble control, miR-214-3p mimics, or si-FRK. (**D**) Representative images of Transwell migration assays in breast cancer cells transfected with scramble control, miR-214-3p mimics, or si-FRK. (**E**) Quantification of cell migration in MCF-10A, MCF-7, and MDA-MB-231 cells. (**F**) Representative images of Transwell invasion assays in MCF-7 and MDA-MB-231 cells transfected with scramble control, miR-214-3p mimics, or si-FRK. (**G**) Quantification of cell invasion in MCF-7 and MDA-MB-231 cells. Data are presented as mean ± standard deviation (SD) from three independent experiments (*n* = 3). * *p* < 0.05, ** *p* < 0.01. Representative images are shown, and all images were acquired under identical magnification and imaging conditions. Quantification was performed from at least three randomly selected fields per condition.

To investigate the functional role of miR-214-3p in breast cancer, cell proliferation assays were performed following transfection with miR-214-3p mimics or si-FRK. Overexpression of miR-214-3p significantly increased cell proliferation in MCF-10A, MCF-7, and MDA-MB-231 cells. Similarly, silencing of FRK also enhanced cell proliferation, indicating that FRK contributes to the effects of miR-214-3p (Figure 3A–C).

Next, Transwell migration assays were conducted to assess the impact of miR-214-3p on cell motility. miR-214-3p overexpression markedly increased the migratory capacity of breast cancer cells. Consistently, FRK knockdown resulted in a similar increase in cell migration (Figure 3D,E).

Furthermore, Transwell invasion assays demonstrated that miR-214-3p significantly enhanced the invasive potential of MCF-7 and MDA-MB-231 cells. In line with these findings, silencing of FRK also increased invasion, suggesting that the pro-invasive effect of miR-214-3p is mediated, at least in part, through suppression of FRK (Figure 3F,G).

Taken together, these results suggest that FRK functions as a downstream mediator of miR-214-3p in regulating breast cancer cell proliferation, migration, and invasion.

## 4. Discussion

In this study, we found that miR-214-3p acts as an oncogenic regulator in breast cancer, particularly in TNBC, by functionally targeting the 3′-UTR of FRK. We showed that miR-214-3p was upregulated in TNBC cells and exhibited a generally inverse trend with FRK expression. Functional assays further revealed that miR-214-3p promoted cell proliferation, migration, and invasion, whereas silencing of FRK recapitulated these effects, supporting a mechanistic link between miR-214-3p and FRK in tumor progression.

Although an inverse relationship between miR-214-3p and FRK expression was observed in some cell lines, this pattern was not consistent across all models. This suggests that FRK expression may be regulated by multiple factors beyond miR-214-3p, reflecting the complexity of post-transcriptional regulation in breast cancer cells. miRNAs operate within complex regulatory networks involving multiple targets and being influenced by additional upstream and downstream modulators. Therefore, the partial inconsistency observed in our data is biologically plausible and consistent with the context-dependent nature of miRNA-mediated regulation.

miR-214-3p has been reported to exert diverse and sometimes conflicting roles in human cancers, acting either as an oncogene or a tumor suppressor depending on tumor type and biological context [9]. In breast cancer, previous studies have shown that miR-214-3p regulates multiple cancer-related pathways through targets such as PTEN, Wnt/β-catenin, and p53 [10,11,12]. These findings indicate that miR-214-3p functions as a central regulator of oncogenic signaling networks rather than acting through a single target. Our results support an oncogenic role for miR-214-3p in TNBC and further suggest that its biological function is subtype-specific, which may partly explain the discrepancies among previous reports.

FRK is a non-receptor tyrosine kinase with context-dependent functions in cancer biology [14]. Although FRK has been reported to exhibit oncogenic properties in certain tumor types, accumulating evidence supports a tumor-suppressive role in breast cancer [14,15]. Mechanistically, FRK has been shown to interact with and stabilize PTEN by inhibiting its ubiquitination, thereby suppressing PI3K/AKT signaling and regulating cell proliferation and survival pathways [15,16]. In this context, downregulation of *FRK* by miR-214-3p may lead to destabilization of PTEN and subsequent activation of oncogenic signaling pathways, providing a plausible mechanistic explanation for the enhanced proliferation, migration, and invasion observed in our study.

An important finding of this study is that silencing of FRK recapitulated the functional effects of miR-214-3p overexpression. This strengthens the biological relevance of the miR-214-3p–FRK axis. However, the magnitude of the effect induced by si-FRK was less pronounced than that observed after miR-214-3p overexpression. This observation is biologically reasonable, as miRNAs typically regulate multiple downstream targets simultaneously. Therefore, although FRK appears to be an important functional mediator of miR-214-3p, additional targets are likely to contribute to the overall oncogenic effect of miR-214-3p in breast cancer.

In this study, we demonstrated that miR-214-3p reduces *FRK* mRNA levels, as demonstrated by qRT-PCR. Although protein-level validation was not included in this study, previous studies suggest that miRNA-mediated repression predominantly occurs through mRNA degradation rather than translational inhibition [18,19]. Together, these findings support a functional regulatory relationship between miR-214-3p and FRK at the post-transcriptional level.

Several limitations of this study should be acknowledged. First, this study is based on in vitro cell line models, and validation in clinical samples will be necessary to confirm the relevance of the miR-214-3p–FRK axis in human breast cancer. Second, although we demonstrated functional targeting of the FRK 3′-UTR by miR-214-3p using luciferase assays, further validation at the protein level would strengthen the mechanistic understanding of this regulatory axis. Finally, because the current work focused on functional validation in cell lines, the prognostic and therapeutic relevance of this axis should be explored in larger translational studies, including analyses using clinical specimens and in vivo models.

## 5. Conclusions

Our findings demonstrate that miR-214-3p contributes to aggressive tumor behavior in breast cancer by targeting FRK. This regulatory interaction provides additional insight into the molecular mechanisms underlying TNBC progression and may offer potential therapeutic opportunities.

## Data Availability

The original contributions presented in this study are included in the article. Further inquiries can be directed to the corresponding authors.
